# Choice of Host Cell Line Is Essential for the Functional Glycosylation of the Fc Region of Human IgG1 Inhibitors of Influenza B Viruses

**DOI:** 10.4049/jimmunol.1901145

**Published:** 2020-01-06

**Authors:** Patricia A. Blundell, Dongli Lu, Anne Dell, Stuart Haslam, Richard J. Pleass

**Affiliations:** * Department of Tropical Disease Biology, Liverpool School of Tropical Medicine, Liverpool L3 5QA, United Kingdom; and; † Department of Life Sciences, Imperial College London, London SW7 2AZ, United Kingdom

## Abstract

Abs are glycoproteins that carry a conserved *N*-linked carbohydrate attached to the Fc whose presence and fine structure profoundly impacts on their in vivo immunogenicity, pharmacokinetics, and functional attributes. The host cell line used to produce IgG plays a major role in this glycosylation, as different systems express different glycosylation enzymes and transporters that contribute to the specificity and heterogeneity of the final IgG-Fc glycosylation profile. In this study, we compare two panels of glycan-adapted IgG1-Fc mutants expressed in either the human endothelial kidney 293-F or Chinese hamster ovary–K1 systems. We show that the types of *N*-linked glycans between matched pairs of Fc mutants vary greatly and in particular, with respect, to sialylation. These cell line effects on glycosylation profoundly influence the ability of the engineered Fcs to interact with either human or pathogen receptors. For example, we describe Fc mutants that potently disrupted influenza B–mediated agglutination of human erythrocytes when expressed in Chinese hamster ovary–K1, but not in human endothelial kidney 293-F cells.

## Key Points

- Choice of cell line is critical for functional glycosylation of human IgG1-Fc.
- Sialylated IgG1-Fc monomers block influenza B only when made by CHO-K1 cells.

## Introduction

In therapeutic approaches in which the Fc of human IgG1 is critically important, receptor binding and functional properties of the Fc are lost after deglycosylation or removal of the Asn-297 *N*-linked glycosylation attachment site located in the body of the Fc (1–3). More detailed studies into the types of sugars involved in this functionality have shown enhanced FcγRIIIA binding and Ab-dependent cellular cytotoxicity of IgG1 in the absence of fucose (4, 5); enhanced FcγRIIIA binding but rapid clearance from the circulation of IgG1 enriched for oligomannose structures (6–8); and improved solubility, anti-inflammatory activity, thermal stability, and circulatory half-life of terminally sialylated glycans from IgG1 (9–13).

These findings have generated an incentive to modify the existing IgG1 glycans attached to Asn-297, either by glycoengineering/chemoenzymatic means (12, 14), by mutagenesis programs on the Fc protein backbone that disrupt the protein–Asn-297–carbohydrate interface (15), or by expression in glycosidase-adapted transgenic cell lines (reviewed in Ref. 16). For example, the Food and Drug Administration–approved humanized Ab mogamulizumab, which is used to treat lymphoma and is manufactured in Chinese hamster ovary (CHO) cell lines in which the α (1–6)–fucosyltransferase (*FUT8*) gene is removed, results in an afucosylated IgG1 with enhanced FcγRIIIA-dependent tumor cell killing by Ab-dependent cellular cytotoxicity (17). Although similar approaches have yielded enhanced sialylation of IgG, with zero to moderate improvements in binding to FcγRs (12, 15, 18, 19), these have not led to significant enhancements in binding to inhibitory glycan receptors that are important in controlling unwanted inflammation (19, 20), a finding we and others have attributed to the buried location of the Asn-297–attached glycan within the Fc (21, 22).

Less is known about glycosylation of the receptors to which the IgG1-Fc binds. Although recent studies implicate an active role for Fcγ receptor–associated carbohydrates in fine-tuning Ab–receptor interactions (23, 24), aglycosylated FcγRs expressed in *Escherichia coli* still retain the ability to bind IgG (25–27), bringing into question the exact role of Fcγ receptor glycosylation to IgG1-Fc binding. Similar arguments apply for most of the glycan receptors. For example, DC-SIGN and DC-SIGNR when expressed by *E. coli* still bind Man_9_GlcNAc_2_ oligosaccharides with high affinity (28). The *N*-linked glycans on glycan receptors are mostly not located near the CRD binding sites but are involved in *cis*-mediated clustering of receptors within the plasma membrane. For example, refolded recombinant *E. coli*–expressed Siglec-5 is sufficient for binding sialylated carbohydrates (29).

We took an alternative approach to enhancing the sialic acid content of the Fc of IgG1 (30, 31) by adding the 18-aa tailpiece from IgM to the C terminus of the IgG1 Fc, into which a cysteine-to-alanine substitution is made at Cys-575, and including an extra *N*-glycosylation site to the N terminus at position Asn-221. The tailpiece also contains an *N*-glycosylation site at Asn-563. This site is known to contain a high proportion of oligomannose glycans that may explain earlier observations of enhanced binding of IgM to mannose-dependent receptors (30–32). When expressed in CHO-K1 cells, these molecules displayed enhanced binding to the low-affinity Fcγ receptors (FcγRIIIA and FcγRIIB), and to multiple glycan receptors that control excessive inflammation by IVIG (30, 31, 33). Two such hypersialylated molecules (D221N/C575A and D221N/C309L/N297A/C575A) also bound recombinant hemagglutinin (HA) from influenza A and B viruses and disrupted influenza A–mediated agglutination of human erythrocytes (31).

CHO cell–based systems remain, by far, the most common mammalian cell line used by the pharmaceutical industry; 84% of products are produced in this cell system, and the remaining approved Abs are produced in either NS0 or Sp2/0 cells (34). Although CHO cells account for the largest number of Food and Drug Administration–approved biotherapeutics (34), they do not express α1,2/3/4 fucosyltransferase and β-1-4-*N*-acetylglucosaminyl-transferase III, which are enzymes expressed in human cells (35). Furthermore, humans have active α2,6-sialyltransferase. As such, CHO-derived IgG1 Fcs are only sialylated through α2,3 linkages, whereas both α2,3 and α2,6 linkages can be found on human IgG1 Fc (30, 35). Previous detailed *N*-glycan structural analysis has confirmed that CHO cells produce only α2,3-sialylated *N*-glycans, whereas human endothelial kidney (HEK) cells produce both α2,3- and α2,6-sialylated *N*-glycans (36, 37). Most nonhuman mammalian cell lines can also attach *N*-glycolylneuraminic acid. Humans do not have an active CMP–*N*-acetylneuraminic acid hydroxylase and, so, do not attach *N*-glycolylneuraminic acid, which can elicit immunogenic responses (35), and consequently, nonhuman cell lines are stringently screened to identify clones that produce proteins with desirable glycan profiles (38).

Human cell lines are a promising alternative to nonhuman cell lines, as they possess fully human posttranslational modifications that reduce downstream processing costs and, more importantly, circumvent any risks associated with immunogenicity from nonhuman glycans. However, human cell lines also have significant limitations, including the capacity to produce sialyl-Lewis^x^, which binds to endothelial selectins in areas of inflammation (39). Although this may potentially be favorable for anti-inflammatory therapies (39), the attached sialyl-Lewis^x^ may also adversely affect the biodistribution and pharmacokinetics of an Fc when used in other clinical contexts, for example, antitumor mAbs. Human cell lines also carry the risk of contamination and forward transmission of human pathogens, in particular viruses, that may explain why CHO-K1 cells are still the preferred cell line used by the pharmaceutical industry (34). These issues led us to compare the functional properties of a panel of Fc mutants generated in CHO-K1 cells with the same set of proteins manufactured by HEK 293-F cells (31).

## Materials and Methods

### Production of mutants

**FIGURE 1.**
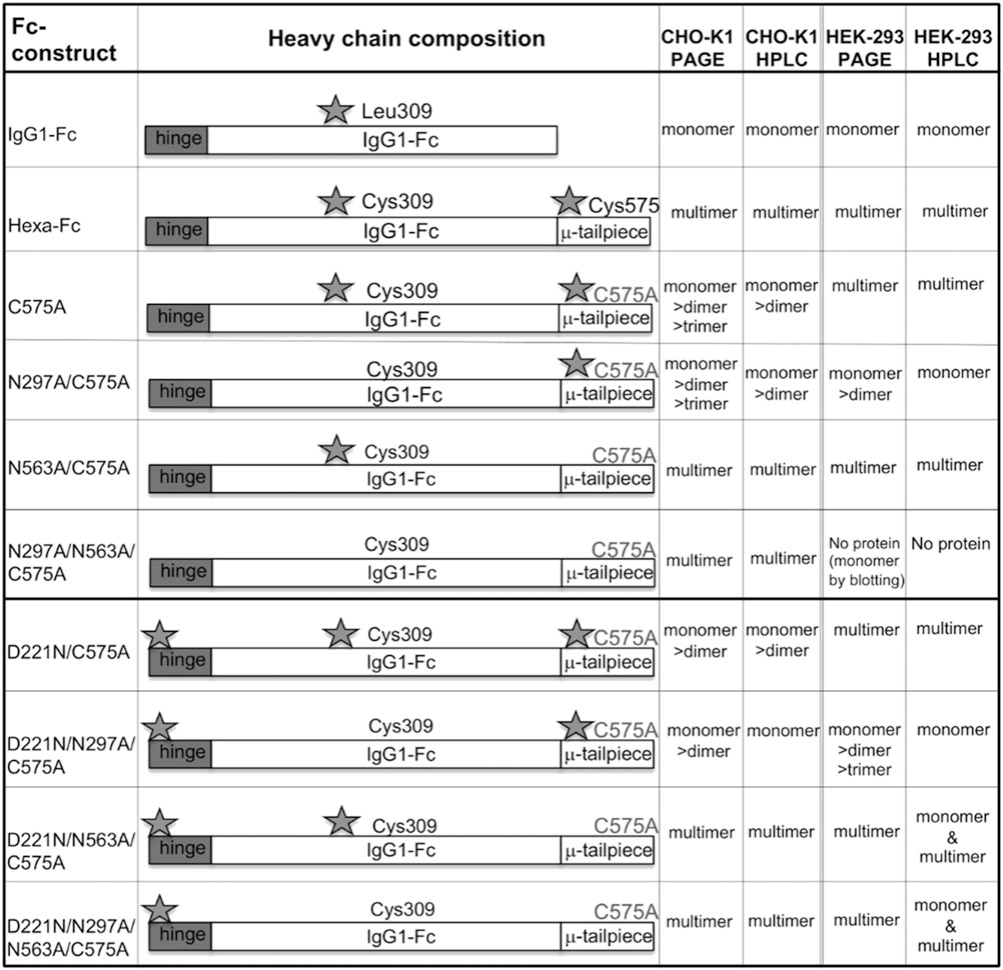
Schematic showing the various HEK 293-F glycan mutants in which Cys-575 is mutated to alanine to create the C575A panel of mutants. Stars indicate the hinge Asn-221, the Cγ2 Asn-297, and the tailpiece Asn-563 glycan sites, respectively.

**FIGURE 2.**
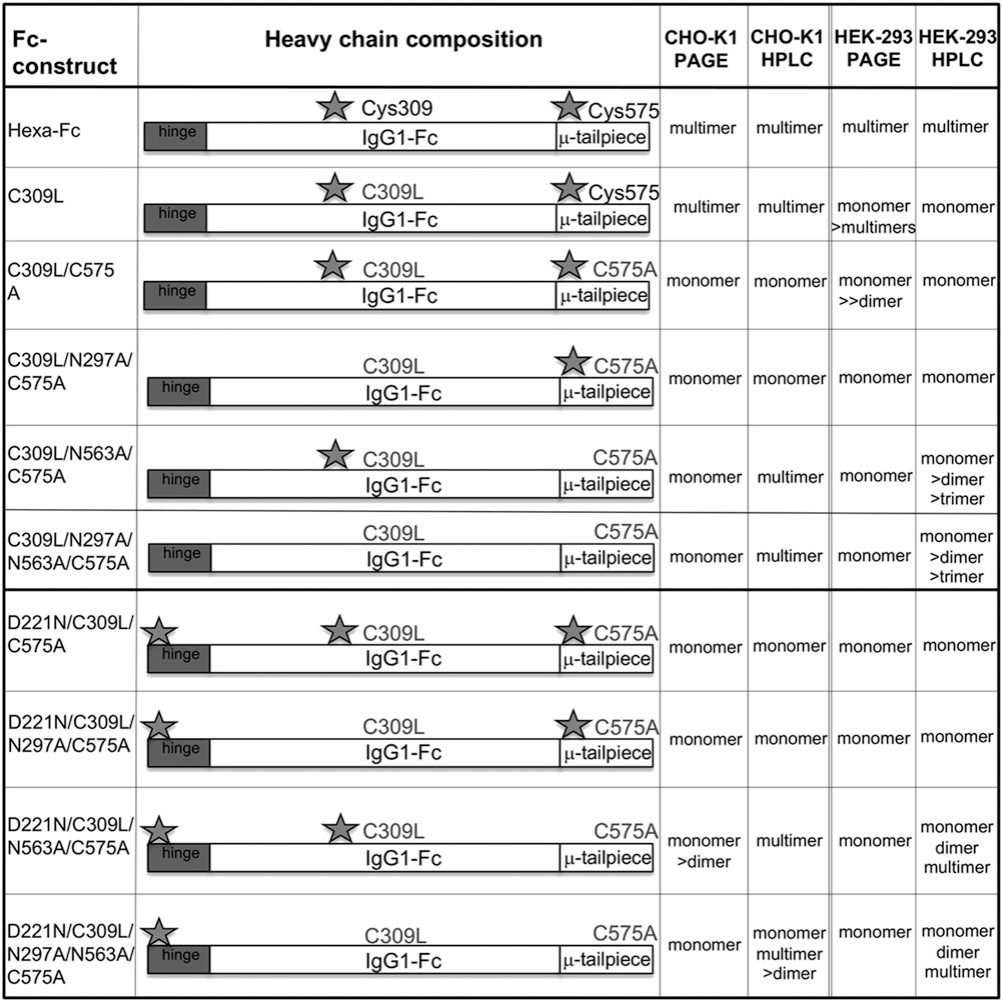
Schematic showing the HEK 293-F C575A panel of glycan mutants from Fig. 1 in which the Cys-309 and Leu-310 are changed to leucine and histidine, as found in the native IgG1 Fc sequence to create the C309L/C575A panel of mutants. Stars indicate the hinge Asn-221, the Cγ2 Asn-297, and the tailpiece Asn-563 glycan sites.

The generation of glycan mutants in all combinations has been described previously for the hexa-Fc that contains cysteines at both positions 309 and 575 (30). To make the new mutants described in Fig. 1 in which Cys-575 was mutated to alanine, PCR overlap extension mutagenesis was used with a pair of internal mismatched primers 5′-ACCCTGCTTGCTCAACTCT-3′/3′-GGCCAGCTAGCTCAGTAGGCGGTGCCAGC-5′ for each plasmid vector coding for a designated glycan modification. The parental plasmids used for these new PCR reactions have been described previously (30). The resulting C575A mutants were then further modified to remove Cys-309 using primer pair 5′-TCACCGTCTTGCACCAGGACT-3′/3′-AGTCCTGGTGCAAGACGGTGA-5′ to create the panel of double cysteine knockouts described in Fig. 2. To verify incorporation of the desired mutation and to check for PCR-induced errors, the open reading frames of the new mutants were sequenced on both strands using previously described flanking primers (30). CHO-K1 cells (European Collection of Cell Cultures) were stably transfected with plasmids using FuGENE (Promega), stable cell lines were created, and Fc-secreting clones were expanded and proteins purified as previously described (22, 40). HEK 293-F cells were transiently transfected using the FreeStyle MAX 293 Expression System (Life Technologies) and proteins purified as for CHO-K1 cells.

### Size analysis using size exclusion HPLC

An SEC 3000 (300 × 7.8 mm) Column (Beckman Coulter) was set up on a Dionex ICS 3000 HPLC System and pre-equilibrated with 0.2-μm-filtered PBS. Protein samples at concentrations ranging from 0.5 to 1 mg/ml were placed in a precooled autosampler at 4°C, and 10 μl of each was sequentially injected onto the column. Each sample was run for 1.5 column volumes in PBS at a flow rate of 0.25 ml/min. Elution was monitored at 280 and 214 nm. The column was calibrated by running standard proteins (thyroglobulin, bovine IgG, OVA, myoglobin, and cyanocobalamin; Bio-Rad Laboratories) under the same conditions.

### Receptor and complement binding assays

Methods describing the binding of mutants to tetrameric human DC-SIGN (Elicityl) and Siglec-1, Siglec-4, and Siglec-3 (Stratech Scientific) have all been described previously (22, 31, 40). The same ELISA protocol was used for Siglec-2, CD23, dectin-1, dectin-2, C-type lectin (clec)–4a, clec-4d, mannose-binding lectin (MBL), and macrophage mannose receptor (Stratech Scientific or Bio-Techne). Binding of C1q has been described previously (22, 31, 40). ELISAs were used to investigate binding of Fc glycan mutants to human FcγRI, FcγRIIA, FcγRIIB, FcγRIIIA, and FcγRIIIB (Bio-Techne). Receptors were coated down onto ELISA plates (Nunc) in carbonate buffer (pH 9) (Sigma-Aldrich) at 2 μg/ml overnight at 4°C, unless otherwise specified. The plates were blocked in PBS/0.1% Tween 20 (PBST) containing 5% dried skimmed milk. Plates were washed three times in PBST before adding Fc mutant proteins at the indicated concentrations and left at 4°C overnight. Plates were washed as above and incubated for 2 h with 1:500 dilution of an alkaline phosphatase–conjugated goat F(ab′)_2_ anti-human IgG (The Jackson Laboratory).

**FIGURE 3.**
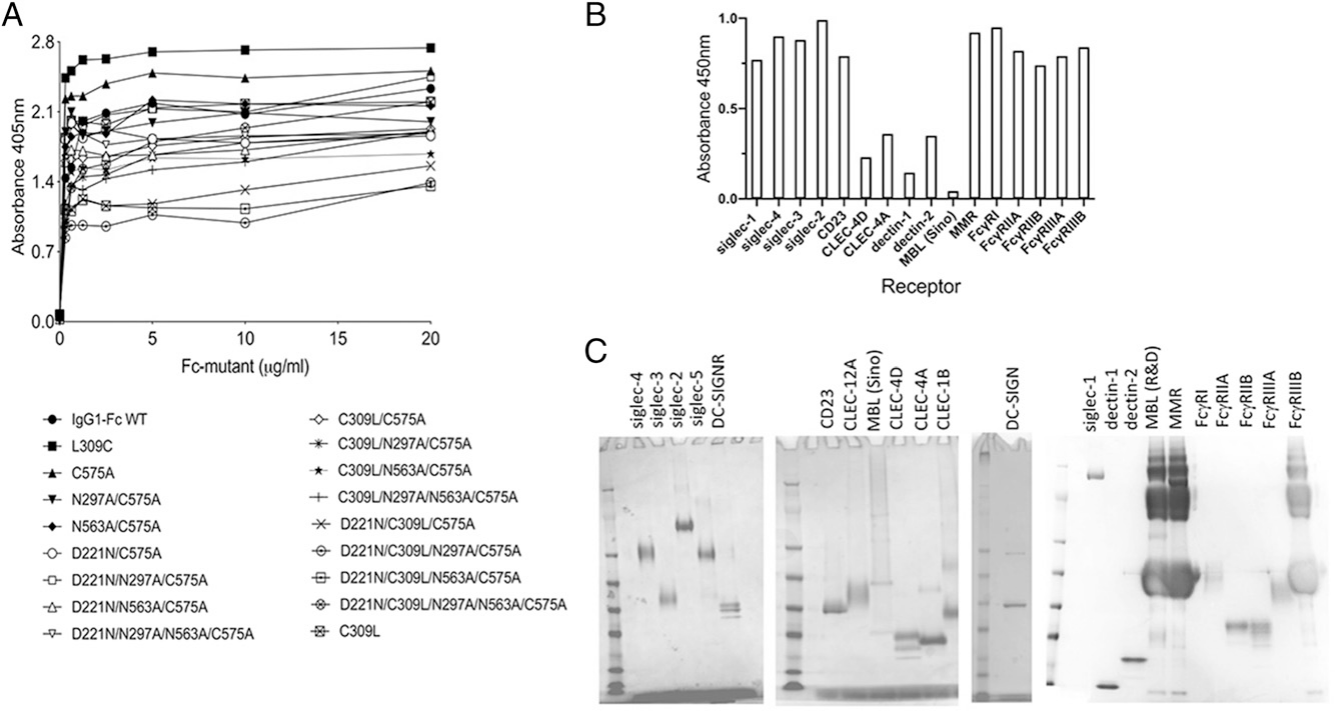
Characterization of detecting reagents and recombinant receptors. (**A**) Characterization of mutant HEK 293-F Fc proteins by direct ELISA. Mutant Fcs were titrated directly onto plates in carbonate buffer overnight. After blocking in PBST 5% skimmed milk, mutants were detected with a 1:1000 dilution of the alkaline phosphatase-conjugated goat F(ab′)_2_ anti-human IgG (The Jackson Laboratory) and developed as per methods. (**B**) Characterization of receptor binding by direct ELISA. The receptors were coated down at 0.2 μg per well in carbonate buffer overnight. After blocking in PBST 5% skimmed milk, mutants were detected with 50 ng/ml of an anti-His mAb (MA1-135; Invitrogen) followed by a 1:4000 dilution of a peroxidase-labeled rat anti-mouse κ (1170-05; Southern Biotech). ELISAs were developed as per methods. (**C**) Receptors were visualized by Coomassie Brilliant Blue staining (as per methods). Two micrograms of each receptor was run on 4–12% SDS-PAGE gradient gels under nonreducing conditions. Note that MBL, macrophage mannose receptor, and FcγRIIIB are sold with BSA as a carrier, which clearly masks the receptor bands.

Binding of the secondary detecting F(ab′)_2_ and levels of Ag coating were checked using either anti-human Fc (Fig. 3A) or anti-histidine (Fig. 3B) Abs, respectively, by direct ELISAs to every mutant and/or receptor to ensure potential biases in the detection of binding of different mutants to different receptors could be considered. Because some of the receptors used are not histidine tagged (e.g., DC-SIGN and MBL) or were poorly recognized by the detecting anti-histidine mAb (e.g., dectin-1, dectin-2, clec-4a, and clec-4d), we also checked protein concentrations prior to coating to plates by SDS-PAGE (Fig. 3C).

Plates were washed and developed for 15 min with 100 μl/well of a SIGMAFAST *p-*Nitrophenyl phosphate solution (Sigma-Aldrich) for alkaline phosphatase–conjugated Abs or with 3,3′,5,5′-tetramethyl-benzidine dihydrochloride (T3405; Sigma-Aldrich) phosphate-citrate buffer (P4922; Sigma-Aldrich) for HRP-conjugated Abs. Plates were read at 405/450 nm and the data plotted with GraphPad Prism.

### Hemagglutination inhibition assay

Native influenza B Hong Kong 5/72 was obtained from Meridian Life Science. To determine the optimal virus-to-erythrocyte ratio, 2-fold virus stock dilutions were prepared in U-shaped 96-well plates (Thermo Fisher Scientific). The same volume of a 1% human O^+^ RBC suspension (Innovative Research) was added to each well and incubated at room temperature for 1 h until erythrocyte pellets had formed. After quantifying the optimal virus-to-erythrocyte concentration (4 HA units), serial 2-fold dilutions of Fc, control IVIG (Gammagard; Baxter Healthcare), and polyclonal goat anti–influenza B (Bio-Rad Laboratories) were prepared, all starting at a concentration of 2 μM, and mixed with 50 μl of the optimal virus dilution. After 30 min incubation at room temperature, 50 μl of the human erythrocyte suspension was added to all wells, and plates were incubated at room temperature for 1 h, after which erythrocyte pellets could be observed in the positive controls and positive samples.

### Binding to FcγRs by biacore

Binding to FcγRs was carried out using a Biacore T200 biosensor (GE Healthcare). Recombinantly expressed FcγRS (R&D Systems or Sino Biological) were captured via their histidine tags onto CM5 chips precoupled with ∼9000 reflective units anti-His Ab (GE Healthcare) using standard amine chemistry. Fc mutants were injected over captured receptors at a flow rate of 20 μl/min, and association and dissociation monitored over indicated time scales before regeneration with two injections of glycine (pH 1.5) and recalibration of the sensor surface with running buffer (10 mM HEPES, 150 mM NaCl [pH 7]). Assays were visualized with Biacore T200 evaluation software v2.0.1.

### *N*-glycomic analysis

*N*-glycomic analysis was based on previous developed protocol with some modifications (41). Briefly, the *N*-glycans from 50 μg of each sample was released by incubation with New England Biolabs Rapid PNGase F and isolated from peptides using Sep-Pak C18 cartridges (Waters). The released *N*-glycans were permethylated prior to MALDI MS analysis. Data were acquired using a 4800 MALDI-TOF/TOF mass spectrometer (Applied Biosystems) in the positive ion mode. The data were analyzed using Data Explorer (Applied Biosystems) and GlycoWorkbench (42). The proposed assignments for the selected peaks were based on composition together with knowledge of biosynthetic pathways.

## Results

### Disulfide bonding and glycosylation influence the multimerization states of Fc mutants expressed by HEK 293-F cells

**FIGURE 4.**
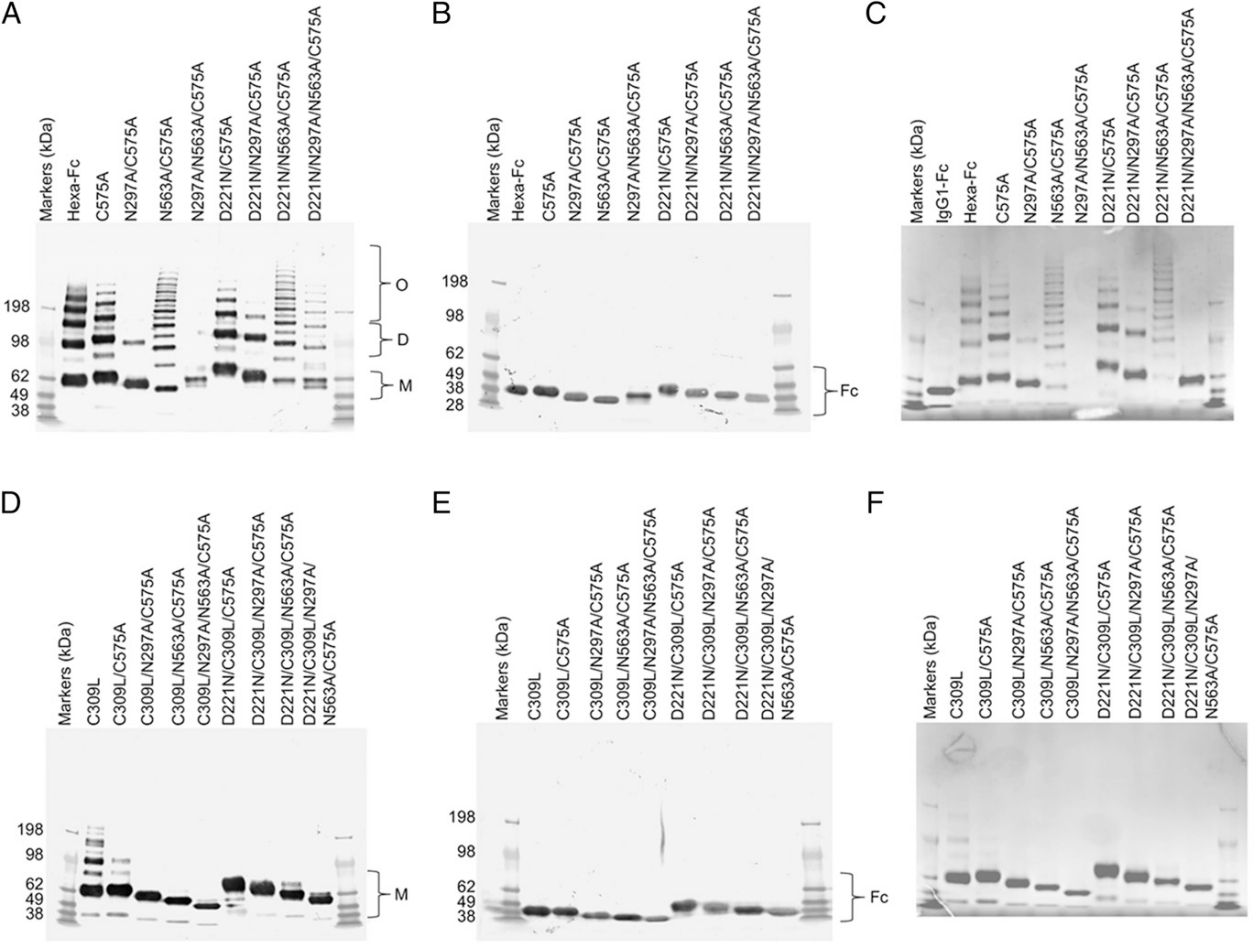
Characterization of mutant HEK 293-F Fc proteins by SDS-PAGE. (**A**) Cys-309 competent mutants in which Cys-575 is mutated to alanine to create the C575A panel of mutants. Mutants with N563A run as laddered multimers. Insufficient material was obtained with N297A/N563A/C575A for further analysis. The addition of the N-X-(T/S) glycan sequon to generate N-terminally glycosylated hinges (the D221N series of mutants) did not affect multimerization but rather increased the molecular mass of all mutants. The N297A mutants run as monomers, dimers, and trimers. (**B**) The same mutants as in (A) but run under reducing conditions. The D221N/C575A mutant has the largest mass, because it has three glycans attached. The types of glycans attached at Asn-221, Asn-297, and Asn-563 for all the mutants are shown in Fig. 9 and Supplemental Figs 1–4. The decreasing molecular masses seen in the Fc represent the sequential loss of *N*-linked glycans. (**C**) The same mutants as in (A) but stained with Coomassie Brilliant Blue reagent. (**D**) Substitution of Cys-309 with leucine onto the C575A mutants shown in (A) to create the double cysteine knockouts, which all run as monomers. C309L in which Cys-575 is present also multimerizes. (**E**) The same mutants as in (D) but run under reducing conditions. Note that the D221N/C309L/C575A mutant with three glycan sites has the largest mass, as seen with the equivalent mutant D221N/C575A in (A). (**F**) Coomassie Brilliant Blue–stained gel of (D). All proteins were run under either nonreducing (A and D) or reducing conditions (B and E) at 2 μg protein per lane on 4–8% acrylamide gradient gels, transferred to nitrocellulose, and for (A), (B), (D), and (E) blotted with an anti-human IgG Fc (Sigma-Aldrich). (C) and (D) are stained with Coomassie Brilliant Blue, showing that only Fc proteins are present after purification. For comparison, the same mutants purified from CHO-K1 cells are shown in (24).

Two panels of glycosylation- and cysteine-deficient mutants previously expressed by CHO-K1 cells were generated in HEK 293-F cells (Figs. 1–3). As observed with CHO-K1 cells, the HEK 293-F cells were capable of making all the mutants to high yields (∼30 mg/l from our limited tissue culture facilities) with the exception of the N297A/N563A/C575A mutant, for which we were unable to generate sufficient protein for further work. Generally, all the mutants migrated on SDS-PAGE with the expected molecular weights for their glycosylation or disulfide bonding states (Fig. 4) and as previously described for the same mutants expressed by CHO-K1 cells (30, 31).

**FIGURE 5.**
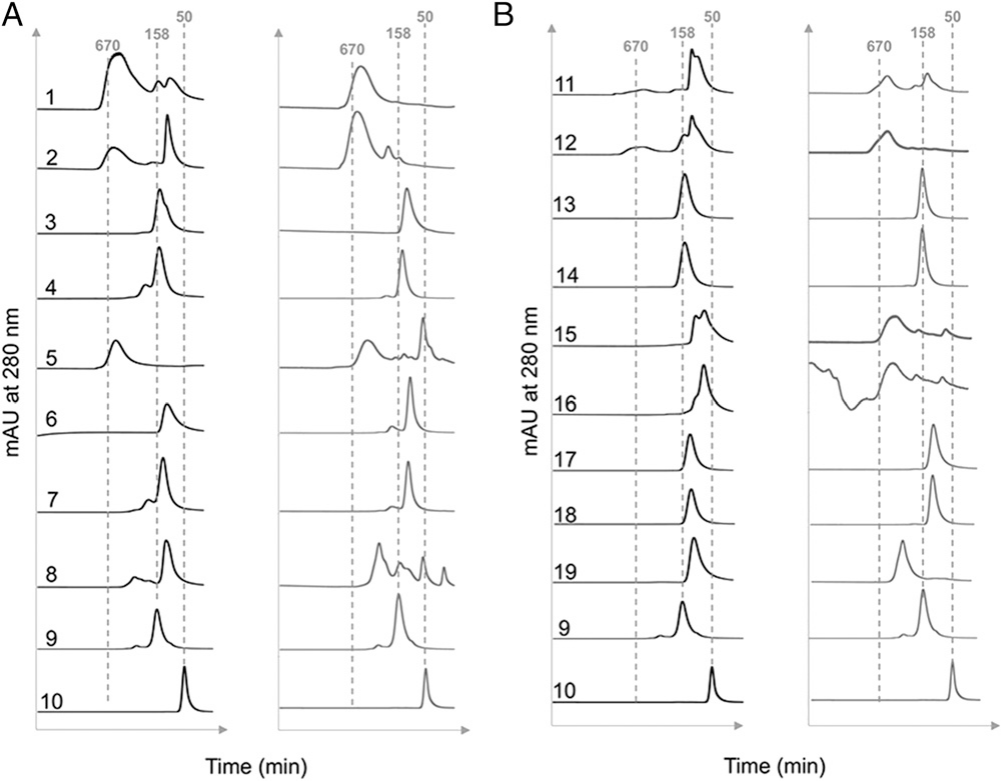
Size-exclusion chromatography analysis of individual Fc mutants expressed by either HEK 293-F (black traces) or CHO-K1 cells (gray traces) as published previously (31). Dotted lines indicate the approximate molecular weights of the control 50 kDa Fc fragment (sample 10) or 158 kDa human IgG (sample 9). The m.w. standards (including 670 kDa thyroglobulin) or the indicated numbered mutants were run and eluted from the column as described in the *Materials and Methods*. (**A**) The C575A panel of mutants: 1, D221N/N297A/N563A/C575A; 2, D221N/N563A/C575A; 3, D221N/N297A/C575A; 4, D221N/C575A; 5, N563A/C575A; 6, N297A/C575A; 7, C575A; 8, Hexa-Fc; 9, IVIG (Gammagard); and 10, IgG1-Fc. (**B**) The C309L/C575A panel of mutants: 11, D221N/C309L/N297A/N563A/C575A; 12, D221N/C309L/N563A/C575A; 13, D221N/C309L/N297A/C575A; 14, D221N/C309L/C575A; 15, C309L/N297A/N563A/C575A; 16, C309L/N563A/C575A; 17, C309L/N297A/C575A; 18, C309L/C575A; and 19, C309L.

In an earlier study with CHO-K1 cells, we demonstrated that a proportion of molecules in which the tailpiece Asn-563 glycan was substituted for alanine ran as multimers in solution when examined by size exclusion HPLC (31). The loss of the bulky Asn-563 glycan exposes hydrophobic amino acid residues in the tailpiece that facilitate noncovalent interactions in solution. Such N563A-dependent multimerization was also observed with mutants expressed by HEK 293-F cells, although the proportion of multimers to monomers (with the notable exception of the N563A/C575A mutant) was generally lower when mutants were made by this cell line (Fig. 5). Clearly, the choice of cell line and, consequently, the types of posttranslational modifications dramatically impact on the biophysical properties of these molecules in solution.

### Fc glycan mutants expressed by HEK 293-F cells show differences in binding to glycan receptors when compared with CHO-K1 proteins

**FIGURE 6.**
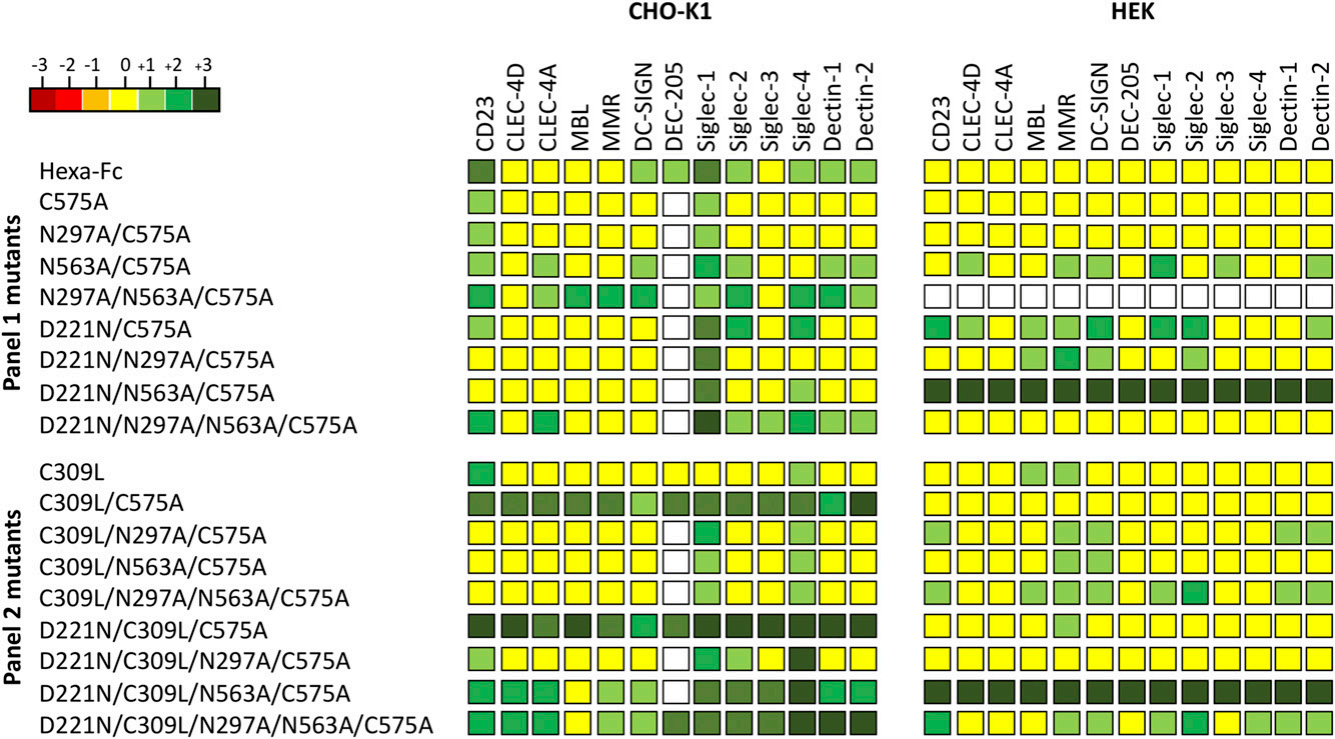
Shading matrix showing the differential binding of HEK 293-F or CHO-K1 mutant proteins to recombinant glycan receptors. Results from at least two independent ELISA experiments are expressed as fold change (up or down) with respect to the internal IgG1 Fc control run on each plate. Standalone ELISA data are provided in the Supplemental Fig. 1. White boxes, not tested.

To determine the impact of the cell line on receptor binding by the two panels of Fc mutants, we investigated their interaction with soluble recombinant glycan receptors by ELISA (Fig. 6, Supplemental Fig. 1). For most of the Fc mutants, including hexa-Fc, C575A, N297A/C575A, D221N/N297A/N563A/C575A, C309L/C575A, D221N/C309L/C575A, D221N/C309L/N297A/C575A, and D221N/C309L/N297A/N563A/C575A, expression in HEK 293-F cells reduced the binding to glycan receptors when compared with equivalent molecules expressed in CHO-K1 cells (Fig. 6). However, two Fc mutants (D221N/N563A/C575A and D221N/C309L/N573A/C575A) were notable for their enhanced and nonspecific binding to all the glycan receptors investigated when expressed in HEK 293-F cells. Given that both mutants multimerize, although not as efficiently as their equivalents made in CHO-K1 cells (Fig. 5), we attribute this enhanced nonspecific glycan receptor binding not only to increased avidity effects, but also to fine differences in the attached glycan structures (Supplemental Figs. 3, 4). Therefore, the choice of cell line can dramatically impact on the ability of individual Fc mutants to interact with glycan receptors.

### Fc glycan mutants expressed by HEK 293-F cells show differences in binding FcγRs receptors compared with CHO-K1 cell proteins

**FIGURE 7.**
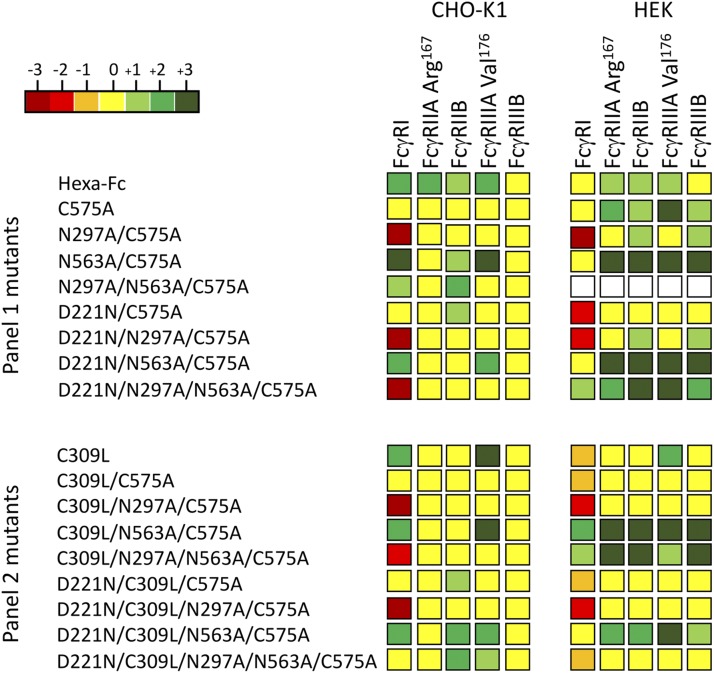
Shading matrix showing the differential binding of HEK 293-F or CHO-K1 mutant proteins to rFcγ receptors. Results from at least two independent ELISA experiments are expressed as fold change (up or down) with respect to the internal IgG1 Fc control run on each plate. Standalone ELISA data are provided in the Supplemental Fig. 2.

Given the observed differences in binding to glycan receptors of the same Fc mutants expressed by two different cell lines, we also tested the impact of cell line on binding to the classical human FcγRs (Fig. 7, Supplemental Fig. 2).

The largest difference observed was the ability of certain HEK-expressed mutants (N563A/C575A, D221N/N563A/C575A, D221N/N297A/N563A/C575A, C309L/N563A/C575A, C309L/N297A/N563A/C575A, and D221N/C309L/N563A/C575A) to bind human FcγRIIA (Arg-167) and FcγRIIIB. This is in stark contrast to the same proteins expressed in CHO-K1 cells, in which not one single mutant from either panel bound the two low-affinity receptors [Fig. 7 and (31)].

**FIGURE 8.**
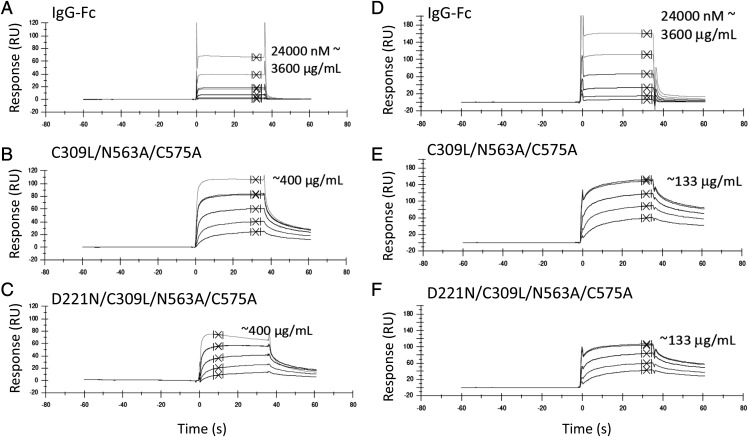
Surface plasmon resonance analysis. Binding of selected mutants to human FcγRIIIB (**A**–**C**) or FcγRIIA-Arg^167^ (**D**–**F**) by Biacore. Control IgG1 Fc (A and D) is compared with C309L/N563A/C575A (B and E) and D221N/C309L/N563A/C575A (C and F). Curves show equivalent molar concentrations doubled diluted from the highest concentration shown. Because of the varying stoichiometry of the molecules shown (as seen in Figs. 3 and 4), an accurate determination of the interaction kinetics is not possible, and *K*_D_ values are calculated assuming a monomeric Fc. Summary tables for the kinetic values obtained from duplicate experiments are shown in Table I. Binding was to receptors sourced from R&D Systems (Bio-Techne).

To examine the interaction with human FcγRIIA (Arg-167) and FcγRIIIB in more detail, we tested binding of two of these mutants (C309L/N563A/C575A and D221N/C309L/N563A/C575A) to FcγRIIIB and FcγRIIA (Arg-167), respectively, by surface plasmon resonance analysis (Fig. 8, Tables I, II). Both mutants displayed slower apparent off rates compared with the control IgG1-Fc monomer, consistent with avidity effects either through binding to multiple immobilized FcγR molecules or rebinding effects (Fig. 8). Therefore, the choice of cell line impacts on the ability of individual Fc mutants to interact with FcγRs and, in particular, FcγRIIA (Arg-167) and FcγRIIIB.

**Table I.** Summary for kinetic data obtained for human FcγRIIIB (CD16B) from SPR analysis

Protein	*K*_D_ (M)*^a^*	R_MAX_	χ^2^ (RU^2^)
Irrelevant IgG1	1.34 × 10^−5^	102.6	0.425
C309L/N563A/C575A	(1) 1.38 × 10^−6^	98.7	9.7
	(2) 2.23 × 10^−6^	113.9	8.39
D221N/C309L/N563A/C575A	(1) 1.53 × 10^−6^	80.6	3.7
	(2) 2.87 × 10^−6^	95.5	3.95

^a^ Proteins were run at the milligram per milliliter equivalent of the Fc control. Curves on each graph in Fig. 8 are approximate equivalent molar concentrations assuming a monomeric Fc. *K*_D_ values are calculated assuming a monomeric Fc. Numbers in parentheses indicate experiment number.

R_MAX_, resonance maximum; RU, response units; SPR, surface plasmon resonance.

**Table II.** Summary for kinetic data obtained for human FcγRIIA_167Arg_ (CD32A) from SPR analysis

Protein	*K*_D_ (M)*^a^*	R_MAX_	χ^2^ (RU^2^)
Irrelevant IgG1	5.98 × 10^−6^	190.1	11.8
C309L/N563A/C575A	(1) 6.20 × 10^−7^	131.9	13.3
	(2) 1.96 × 10^−6^	61.2	2.62
D221N/C309L/N563A/C575A	(1) 6.62 × 10^−7^	92.9	1.38
	(2) 1.75 × 10^−6^	34.1	0.49

^a^ Proteins were run at the milligram per milliliter equivalent of the Fc control. Curves on each graph in Fig. 8 are approximate equivalent molar concentrations assuming a monomeric Fc. *K*_D_ values are calculated assuming a monomeric Fc. Numbers in parentheses indicate experiment number.

SPR, surface plasmon resonance.

### Fc glycan mutants expressed by HEK 293-F cells show improved binding to human C1q

**FIGURE 9.**
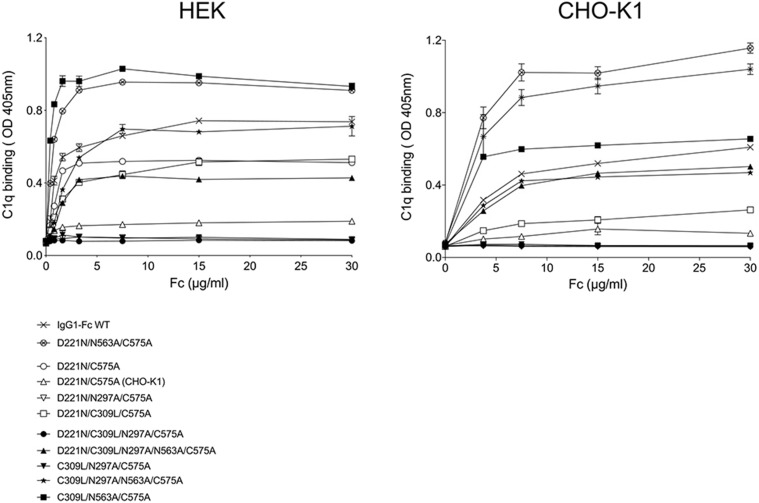
Binding of selected C575A and C309L/C575A mutants to complement component C1q. Mutants expressed in HEK 293-F cells bind human C1q better than the equivalent mutants expressed in CHO-K1 cells. Compare for example the D221N/C575 mutant made in CHO-K1 cells (open triangle) against the same mutant made in HEK 293-F cells (open circle) and compared on the same plate. Error bars represent SD around the mean value (*n* = 2 independent ELISA experiments).

An important functional and safety attribute for therapeutic administration of Fc fragments is their ability to bind C1q and, thus, initiate the classical pathway of complement activation. Binding of C1q was assessed by ELISA to selected mutants expressed from each cell line (Fig. 9). Mutants D221N/C575A, D221N/C309L/C575A, C309L/N297A/N563A/C575A, and C309L/N563A/C575 expressed in HEK 293-F cells showed improved binding to C1q, compared with their counterparts expressed in CHO-K1 cells, and no change in binding in either direction was observed for IgG1-Fc, D221N/N563A/C575A, D221N/N297A/C575A, D221N/C309L/N297A/C575A, D221N/C309L/N297A/N563A/C575A, and C309L/N297A/C575A (Fig. 9).

**FIGURE 10.**
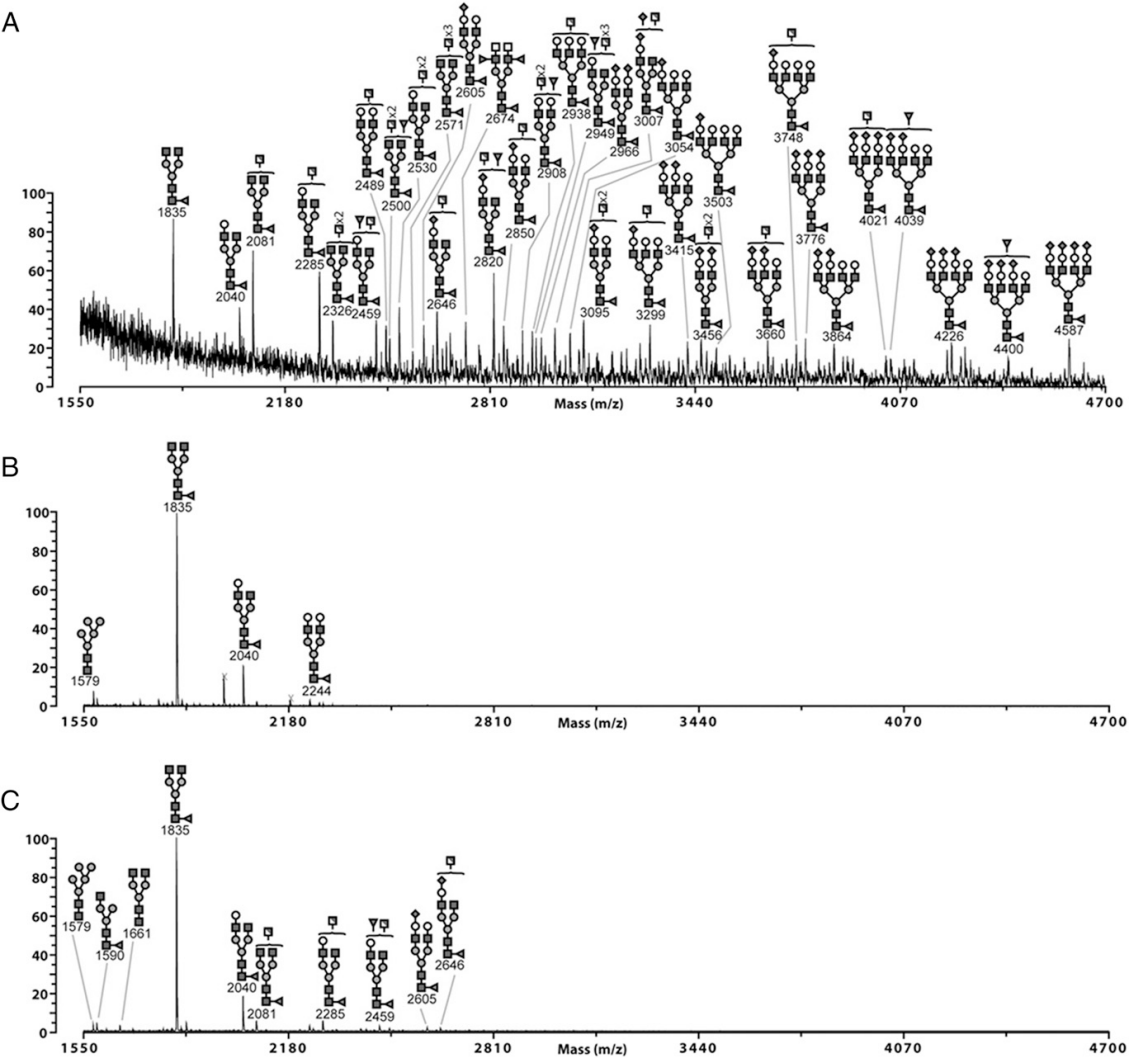
MALDI-TOF MS profiles of permethylated *N*-glycans from the N297A/C575A (Asn-563) (**A**), N563A/C575A (Asn-297) (**B**), and D221N/N297A/N563A/C575A (Asn-221) (**C**) Fc glycan mutants expressed in HEK 293-F cells. Linkage-determined monosaccharides are positioned above the bracket on a structure. Poly-hexose contaminants are highlighted with crosses. The data were acquired in the positive ion mode to observe [M^+^Na]^+^ molecular ions. All the structures are based on composition and knowledge of *N*-glycan biosynthetic pathways. Structures shown outside a bracket have not had their antenna location unequivocally defined.

**FIGURE 11.**
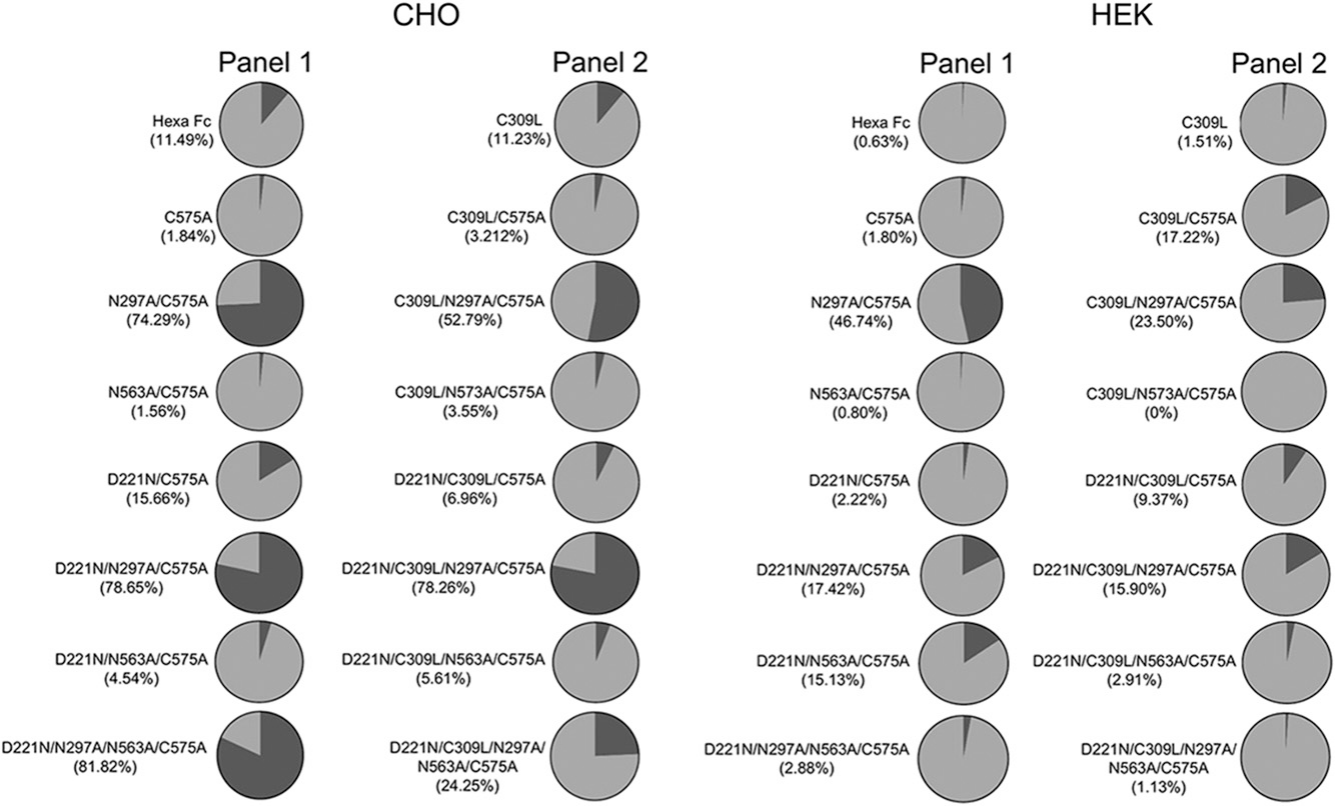
Semiquantitative determination of sialylated (black) against neutral (gray) glycans from the C575A and C309L/C575A mutants expressed in CHO-K1 or HEK 293-F cells. Values shown in brackets under the names of each mutant show percentage-sialylated structures as determined from summed intensities.

**FIGURE 12.**
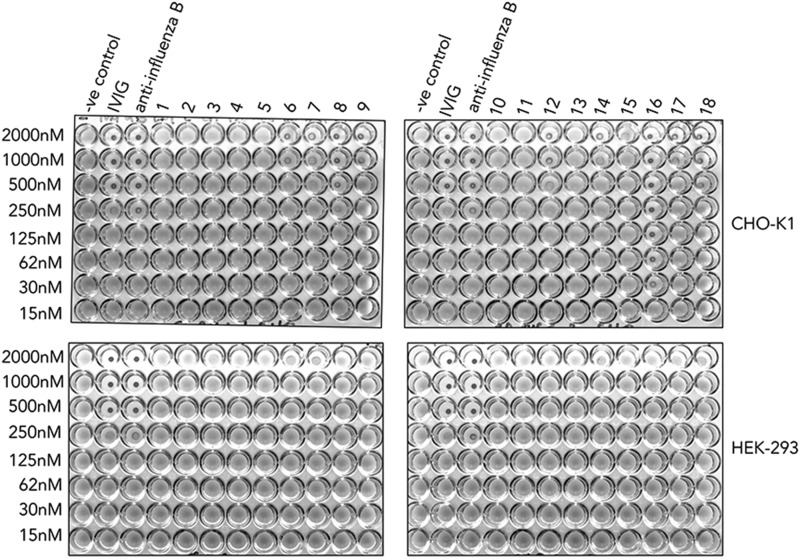
Impact of Fc glycosylation on influenza B–mediated hemagglutination. Mutant Fcs manufactured in either HEK 293-F or CHO-K1 cells were compared with equimolar concentration of IVIG or polyclonal anti–influenza B Abs at inhibiting virus-mediated agglutination of human erythrocytes. 1, L309C; 2, C575A; 3, N297A/C575A; 4, N563A/C575A; 5, no protein; 6, D221N/C575A; 7, D221N/N297A/C575A; 8, D221N/N563A/C575A; 9, D221N/N297A/N563A/C575A; 10, C309L; 11, C309L/C575A; 12, C309L/N297A/C575A; 13, C309L/N575A/C575A; 14, C309L/N297A/N563A/C575A; 15, D221N/C309L/C575A; 16, D221N/C309L/N297A/C575A; 17, D221N/C309L/N563A/C575A; and 18, D221N/C309L/N297A/N563A/C575A. A constant amount of influenza B Hong Kong 5/72 virus was incubated with titrated amounts of the Fc glycan mutants and added to human O^+^ erythrocytes that were then allowed to sediment at room temperature for 1 h. Nonagglutinated RBCs form a small halo (*n* = 3 independent experiments).

Both the D221N/C575A and D221N/C309L/N297A/C575A mutants from CHO-K1 cells have been shown previously to block influenza-mediated hemagglutination [(24) and Figs. 10–12, below], and thus, D221N/C575A expressed in HEK 293-F cells that binds C1q may not be favored for clinical development over the same molecule expressed by CHO-K1 cells (35).

### Fc glycan mutants expressed in HEK 293-F cells have more complex glycosylation profiles than the equivalent mutants expressed in CHO-K1 cells

The structure of the *N*-glycans on the Fc of IgG Abs has been shown to influence multiple receptor interactions (3, 43, 44). Unlike the relatively simple glycosylation of the Fc mutants previously described for CHO cells (30, 31), HEK cells are capable of producing more complex *N*-glycan structures on their glycoproteins (45).

We investigated the nature of the *N*-glycans on the two panels of glycosylation- and cysteine-deficient mutants by MALDI-TOF mass spectrometry–based glycomic analysis (complete dataset for both panels of mutants provided in Supplemental Figs. 3, 4). A core-fucosylated biantennary structure without antennary galactosylation, *m/z* 1835 (GlcNAc_4_Man_3_Fuc_1_), is the base peak of spectra from all IgG1-Fc mutants produced by HEK cells (Fig. 10, Supplemental Figs. 3, 4).

An indication of the types of glycans attached to either Asn-221, Asn-297, or Asn-563 could be determined using both the C575A or the C309L/C575A panels of mutants. For example, only *N*-glycans attached to Asn-297 are available for sampling in either the N563A/C575A or C309L/N563A/C575A, mutants that, therefore, also allow the contribution of disulfide bonding to glycosylation at Asn-297 to be elucidated.

In these mutants, *N*-glycosylation of Asn-297 is dominated by core-fucosylated biantennary glycans (*m/z* 1835 and 2040) with varied galactosylation levels (Gal_0–2_GlcNAc_4_Man_3_Fuc_1_), and a Man_5_GlcNAc_2_ (*m/z* 1579) oligomannose structure is also observed (Fig. 10B). The Asn-563 *N*-glycans are much more complex and heterogeneous. Abundant truncated structures at *m/z* 2081 and 2285 have potentially terminal *N*-acetylglucosamine (GlcNAc) or *N*-acetylgalactosamine (GalNAc) (Fig. 10A). Antennal fucosylation and sialylation are also observed on structures that can assemble sialyl–*N*-acetyllactosamine, sialyl-Lewis x/a, fucosylated LacdiNAc, or sialylated LacdiNAc (GalNAc–GlcNAc), for example, peak *m/z* 4039 (NeuAc_2_Gal_4_GlcNAc_6_Man_3_Fuc_2_). The presence of *m/z* 2674 (GalNAc_2_GlcNAc_4_Man_3_Fuc_3_) in the N297A/C575A mutant confirms the presence of fucosylated LacdiNAc epitopes on the Asn-563 site. Thus, glycosylation at Asn-563 is different to that seen from CHO-K1 cells that assemble less-diverse structures without antennal fucosylation and, therefore, more terminal sialyl–*N*-acetyllactosamine (30, 31). In agreement with earlier work (32) and irrespective of the cell line used for their manufacture, a high proportion of oligomannose structures can be found attached to Asn-563 (e.g., in the C309L/N297A/C575A mutant) (Supplemental Fig. 4), although these were not observed in the corresponding N297A/C575A mutant (Fig. 10A). We do not have an obvious explanation for these Asn-563 differences, although the presence of redox-sensitive cysteines are known to modify protein glycosylation in other systems (46).

The Asn-221 site is mainly composed of biantennary complex structures (Fig. 10C). Excluding the base peak, four structures in the C575A background (*m/z* 2081, 2285, 2459, and 2646) or five structures in the C309L/C575A background (2081, 2285, 2459, 2489, and 2734) could form LacdiNAc antenna (GalNAc–GlcNAc). Antennal fucosylation and sialylation is also observed (Fig. 10C, Supplemental Fig. 4).

In summary, these data show that the types of glycans attached to either Asn-221, Asn-297, or Asn-563 are different between cell lines but are not grossly affected by disulfide bonding.

### Fc glycan mutants expressed in HEK 293-F cells are less sialylated than the equivalent mutants expressed in CHO-K1 cells

Site-specific levels of sialylation were semiquantitatively assessed for both panels of mutants and compared with levels seen in the equivalent mutants expressed in CHO-K1 cells (Fig. 11). Although levels of sialylated glycans attached at positions Asn-297 (the N563A/C575A mutant) and Asn-563 (the N297A/C575A mutant) are similar for both cell lines (Fig. 11), a marked reduction in levels of sialylated glycans at Asn-221 (the D221N/N297A/N563A/C575A mutant) is observed when this mutant is expressed in HEK cells (2.8% against 81.8% in CHO; Fig. 11). Removal of Asn-297 generally enhanced levels of sialylation at both Asn-221 and Asn-563, irrespective of the cell line or the multimerization state of the proteins (e.g., compare N297A/C575A versus C575A and C309L/N297A/C575A versus C309L/C575A) (Fig. 11). The choice of cell line, therefore, dramatically affects the overall levels of sialylation at individual *N*-linked attachment sites within the glycan-modified Fc variants.

### Asn-221–containing mutants are poor inhibitors of hemagglutination by influenza virus when expressed in HEK 293-F cells

To test if the choice of cell line affected the functionality of the two panels of mutant Fcs, we used the World Health Organization hemagglutination inhibition assay (HIA) to quantify the inhibitory titers for each mutant against an influenza B virus (Fig. 12). As shown previously with an avian influenza A (H1N1) (31), mutants containing Asn-221 hinge–attached glycans, and, in particular, the D221N/C309L/N297A/C575A mutant, prevented hemagglutination by an influenza B virus at concentrations as low as 30 nM, an 8-fold improvement over equimolar IVIG or polyclonal anti–influenza B antisera (Fig. 12). In stark contrast, the same mutants expressed by HEK 293-F cells were unable to inhibit hemagglutination by either influenza A (data not shown) or influenza B virus (Fig. 12). This shows that the functional potential of individual glycan-modified Fc mutants is dependent on the choice of cell line used for their manufacture.

## Discussion

We have shown using CHO-K1 cells that the structure and effector function of human IgG1-Fc can be profoundly altered by the addition or removal of *N*-linked glycosylation (30, 31). For example, we could show that Fc fragments containing complex biantennary glycans attached to both the N- and C-terminal ends of the Fc could inhibit influenza A–mediated agglutination of human erythrocytes (31). The aim of the current study was to reveal possible variation in functional glycosylation related to differences in two host cell lines, CHO-K1 and HEK 293-F, particularly as Abs and Fc fusions are the fastest growing therapeutic class in the pharmaceutical industry (34, 47, 48).

Two intriguing aspects of *N*-linked glycosylation are relevant to this study. First, the differential binding seen to human glycan (Fig. 6) and Fcγ (Fig. 7) receptors between the same mutants expressed by two different cell lines. These differentially manufactured mutants now need to be compared in relevant in vivo disease models in which the Fc is therapeutically useful, given that differential sialic acid linkages, α2,6 and α2,3, are known to impact on the anti-inflammatory properties of the Fc (49, 50). Such nuanced glycosylation may also explain why the therapeutic efficacy of molecules generated by different expression systems, and subsequently tested in different animal models, do not always translate to efficacy in human studies (51).

Second, we have studied the exquisite impact of the host cell line on the efficacy of sialylated Fcs to inhibit influenza viruses (Fig. 12). One possible explanation is that overall sialylation levels for all the influenza blocking mutants, in particular the D221N/C309L/N297A/C575A mutant, are ∼5-fold lower when expressed by HEK 293-F cells (Fig. 11). However, overall level of sialylation is not the only possible explanation for the relative efficacy of the CHO-K1 mutants in inhibiting influenza virus hemagglutination, as the CHO-K1–expressed D221N/C575A mutant also contained ∼5-fold-less sialyation than the D221N/C309L/N297A/C575A mutant made in the same cell line (Fig. 11). This indicates that the fine specificity (e.g., α2,3 versus α2,6 linkages) of these sialylated glycans may also be a contributing factor to their efficacy.

As demonstrated previously for influenza A (31), binding and inhibition of influenza B viruses is stronger with mutants containing Asn-221 and, in particular, by the monomeric mutant D221N/C309L/N297A/C575A in which the N- and C-terminal sialylated sugars are spaced ∼60 Å apart [Fig. 12 and (31)]. Recent biophysical studies with alternative glycan–decorated scaffolds have shown that ∼1000-fold enhancements over monovalent binding to HA can be achieved with only two sialylated ligands, provided the sugars are arranged 50–100 Å apart (52, 53).

As observed with Fc-multimerizing mutants from panel 1, no additional benefit with respect to virus neutralization was gained with larger, more complex sialylated structures (Fig. 12, and as seen with the D221N/N297A/N563A/C575A and D221N/N563A/C575A mutants). The lack of monomers observed for both these mutants when expressed by HEK cells (Fig. 5A) may offer a simpler explanation for why the HEK-expressed proteins did not inhibit influenza viruses in the HIA, yet their equivalents expressed by CHO-K1 cells and that contain a large proportion of monomers did partially inhibit hemagglutination by virus (Fig. 12). As multimers, neither of the sialylated Fc-capping glycans (Asn-221 or Asn-563) may be optimally available for interactions with HA as they are with mutants that uniquely exist as monomers (e.g., D221N/C309L/N297A/C575A) (30, 31).

We do not yet know if sialylated Fcs are susceptible to cleavage by the viral neuraminidase (NA). Although a decoy for NA may be a therapeutically attractive strategy (54), we have not observed a direct decay in the HIA after prolonged incubation. This suggests that the high specific avidity of these molecules for HA may reduce their susceptibility to NA, a hypothesis that fits with the relatively low efficiency of NA (*k*_cat_ = 30–155^s−1^), together with the asymmetric distribution of NA in relation to HA on the surface of filamentous influenza viruses (55–57).

To be useful compounds when administered intranasally, or as an aerosol, the sialylated Fc needs to outcompete sialylated mucins that viruses use through ratchet-like interactions with HA and NA to migrate to the underlying respiratory epithelium (55). Of the 15 known human mucins in the human lung, only MUC5 has been shown to protect from influenza (58, 59). Most sialic acid found on human mucins are *O*-glycosylated, and where *N*-linked attachments do occur, these are mostly sialylated via α2,6-linkages (59). Thus, we were surprised that none of the Fc leads inhibited influenza A (H1N1 propagated in hen eggs) or influenza B (Hong Kong 5/72 propagated in Madin–Darby canine kidney cells) agglutination of human O^+^ erythrocytes when manufactured by HEK 293-F cells that attach more human type α2,6-linked sialic acid (Fig. 10).

The apparent importance of α2,3-linked *N*-glycans to inhibition of both influenza A and B by the CHO-K1 Fc mutants indicates that viruses can evolve away from inhibition by mucus whose predominant *O*-linked glycans are mostly α2,6-linked. Our working hypothesis is that HEK-expressed compounds may therefore inhibit influenza viruses that circulate in human populations or that are propagated in cell lines that attach more human-like α2,6-linked sialic acid.

Consequently, by careful consideration of the cell line used in their manufacture, new glycan repertoires with desirable binding attributes and functionality can be imparted to the therapeutically attractive Fc molecule.

## Supplementary Material

Data Supplement
