# Supplementary material for: Choice of Host Cell Line Is Essential for the Functional Glycosylation of the Fc Region of Human IgG1 Inhibitors of Influenza B Viruses
Source: J Immunol. 2020 Jan 6;204(4):1022–34. doi: 10.4049/jimmunol.1901145 (PMC6994840; doi:10.4049/jimmunol.1901145)
Supplement: Data Supplement [file JI_1901145.zip › JI_1901145_Supplemental_Figures_1.pdf]

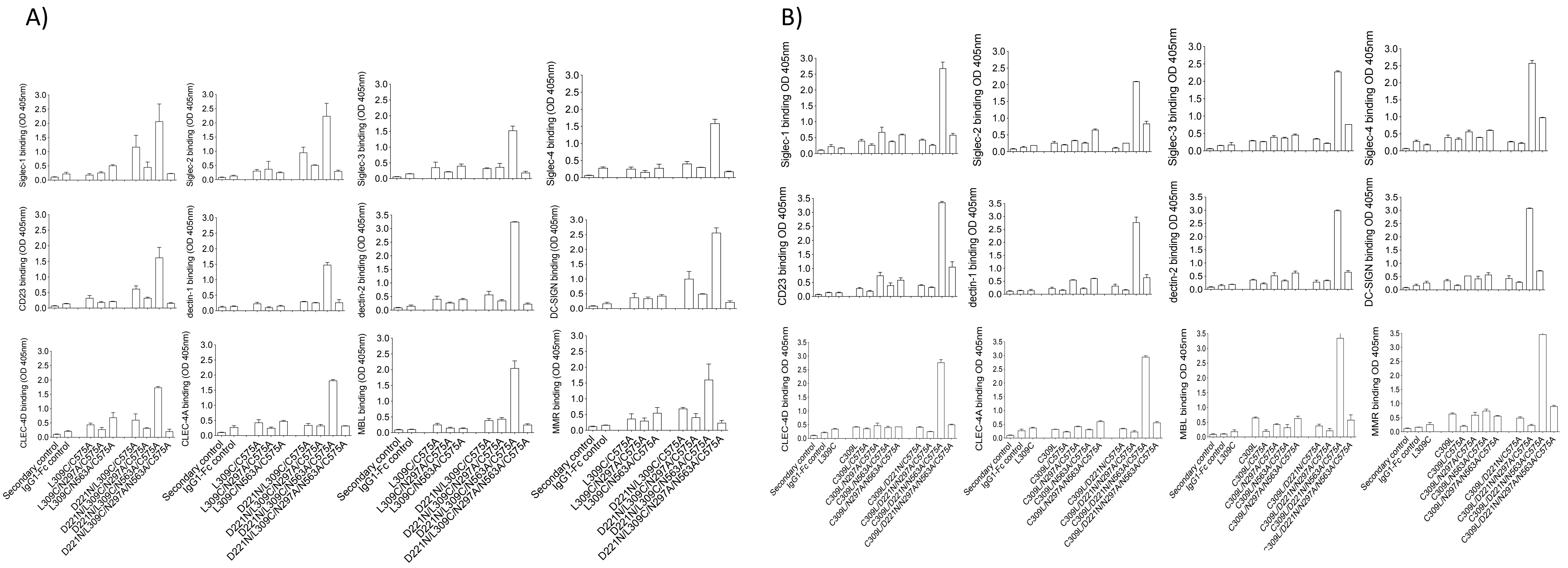

FIGURE S1. Glycan receptor binding data for series 1 (A) and series 2 (B) mutants.
Series 1 and 2 mutants are shown in Figs. 1 and 2 respectively and manufactured as described in methods.
Error bars represent standard deviations around the mean value; n = 2 independent experiments.

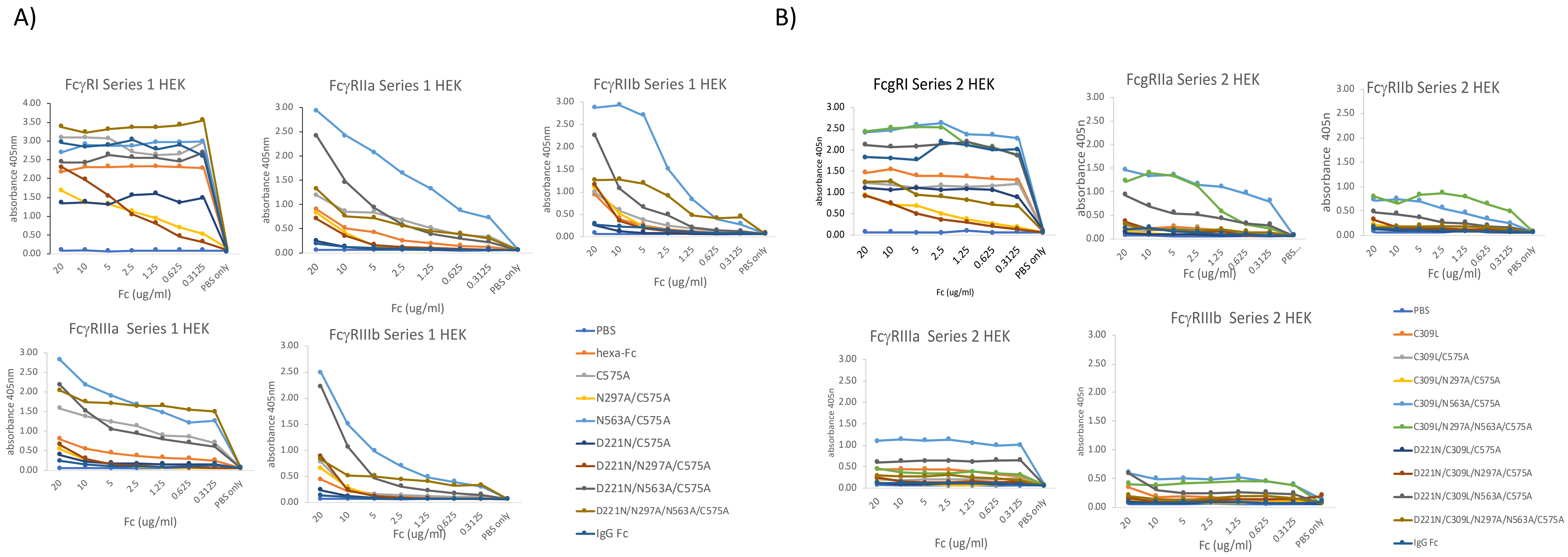

FIGURE S2. Fcγ-receptor binding data for series 1 (A) and series 2 (B) mutants.
Series 1 and 2 mutants are shown in Figs. 1 and 2 respectively and manufactured as described in methods.
Error bars represent standard deviations around the mean value; n = 2 independent experiments.

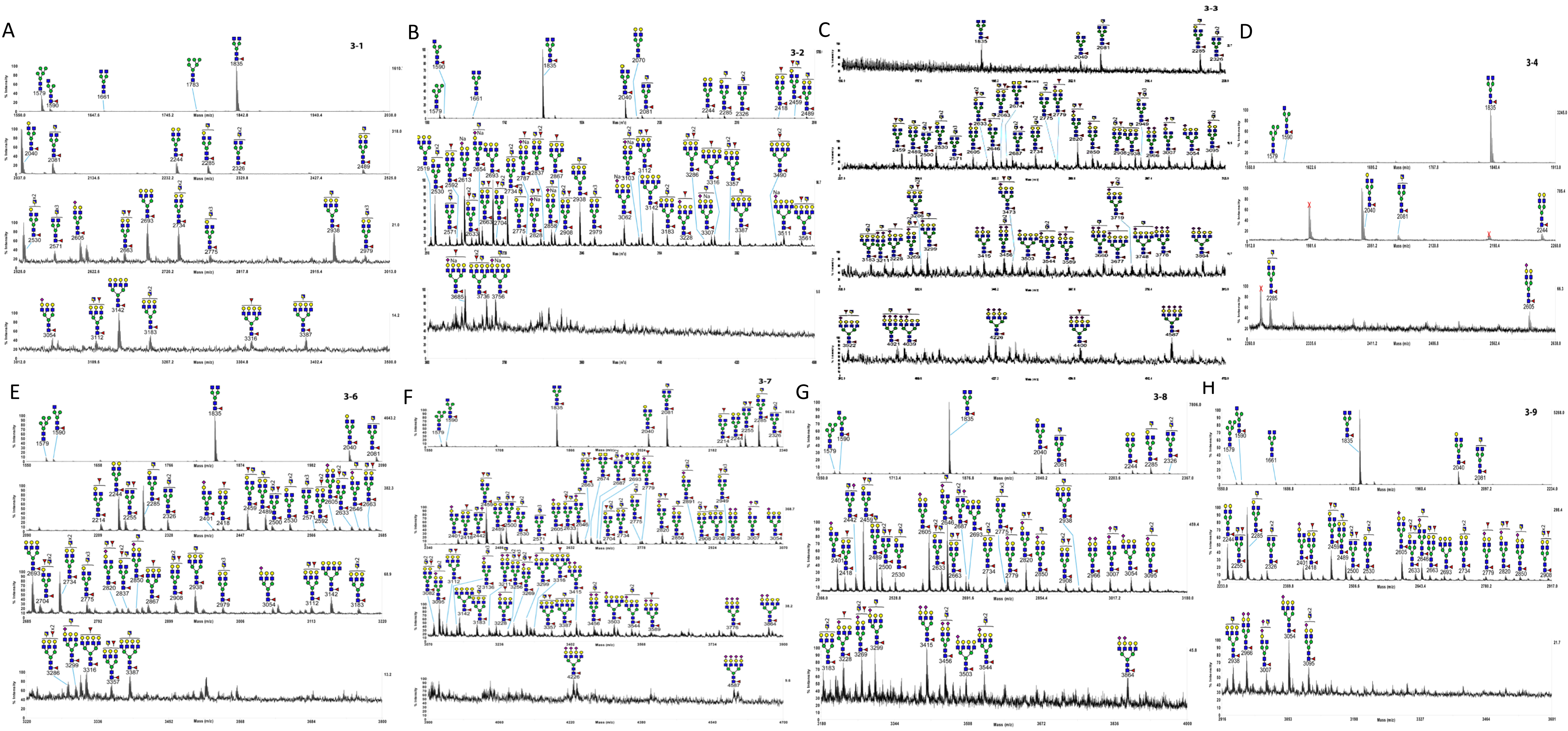

FIGURE S3. MALDI-TOF MS profiles of permethylated *N*-glycans from series 1 glycan mutants expressed in HEK 293-F cells. Linkage determined monosaccharides are positioned above the bracket on a structure. Data were acquired in positive ion mode to observe $[M+Na]^+$ molecular ions. All structures are based on composition and knowledge of N-glycan biosynthetic pathways. Structures shown outside a bracket have not had their antenna location unequivocally defined. Poly-hexose contaminants are highlighted with crosses. (A) C309L/N297A/C575A, (B) C575A, (C) N297A/C575A, (D) N563A/C575A, (E) D221N/C575A (F) D221N/N297A/C575A, (G) D221N/N563A/C575A, and (H) D221N/N297A/N563A/C575A

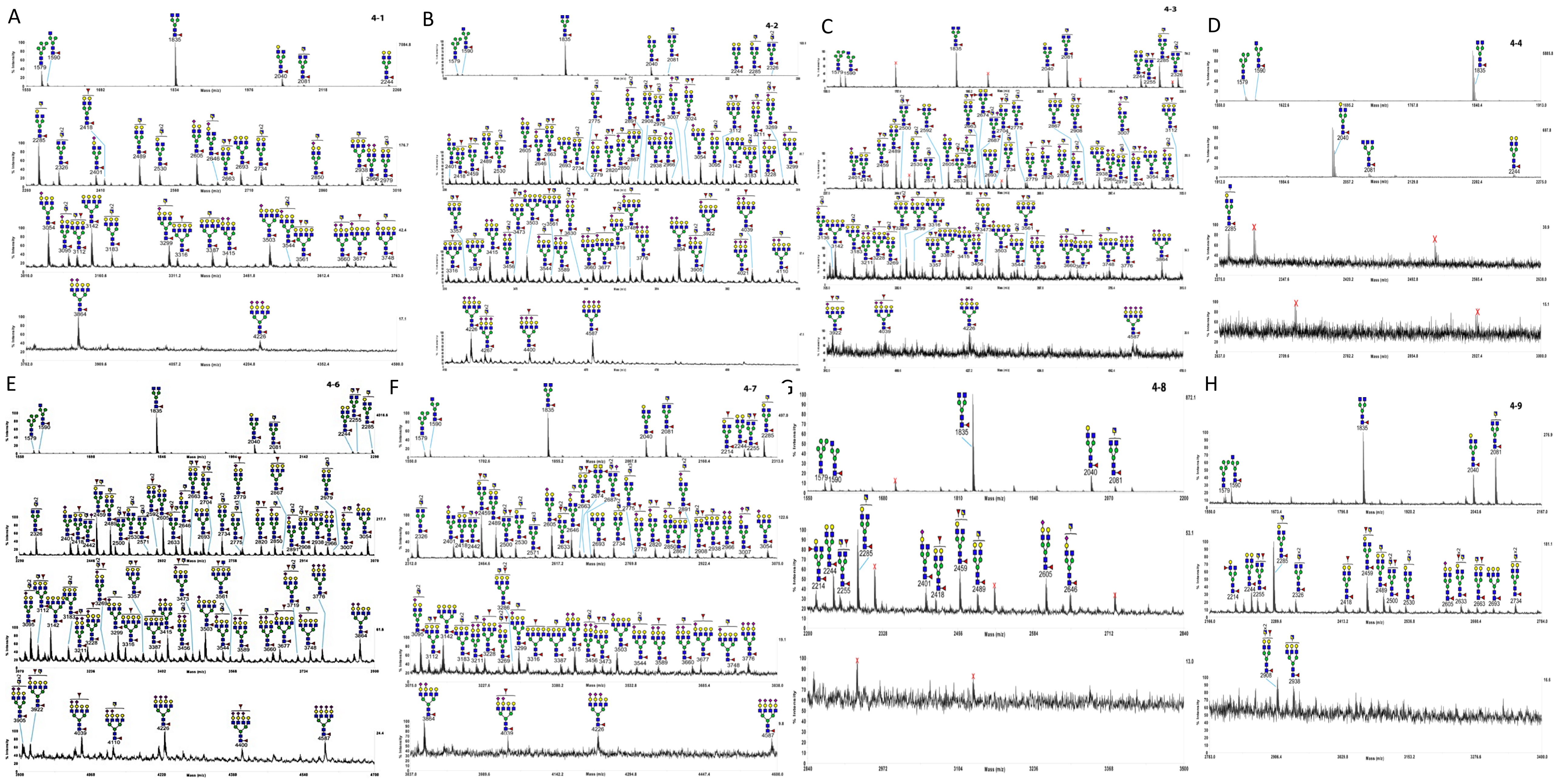

FIGURE S4. MALDI-TOF MS profiles of permethylated *N*-glycans from series 2 glycan mutants expressed in HEK 293-F cells. Legend descriptor as per Fig. S3. (A) C309L, (B) C309L/C575A, (C) C309/N297A/C575A, (D) C309L/N563A/C575A, (E) D221N/C309L/C575A, (F) D221N/C309L/N297A/C575A, (G) D221N/C309L/N563A/C575A, and (H) D221N/C309L/N297A/N563A/C575A. No glycans were detected from the C309L/N297A/N563A/C575A mutant.
